# Interruption of Jasmonic Acid Biosynthesis Causes Differential Responses in the Roots and Shoots of Maize Seedlings against Salt Stress

**DOI:** 10.3390/ijms20246202

**Published:** 2019-12-09

**Authors:** Ramala Masood Ahmad, Cheng Cheng, Jia Sheng, Wei Wang, Hong Ren, Muhammad Aslam, Yuanxin Yan

**Affiliations:** 1 State Key Laboratory of Crop Genetics and Germplasm Enhancement, Nanjing Agricultural University, Nanjing 210095, China; 2016201089@njau.edu.cn (R.M.A.); 2016201062@njau.edu.cn (C.C.); 2017101141@njau.edu.cn (J.S.); 2 Jiangsu Collaborative Innovation Center for Modern Crop Production, Nanjing 210095, China; 3 Guizhou Institute of Upland Food Crops, Guizhou Academy of Agricultural Sciences, Guiyang 550006, China; ww1980666@126.com (W.W.); rhong666@163.com (H.R.); 4 Department of Plant Breeding and Genetics, University of Agriculture Faisalabad, Faisalabad 38000, Pakistan; aslampbg@gmail.com

**Keywords:** jasmonate, salt response, *Zea mays*, ROS, proline, ABA biosynthesis

## Abstract

Jasmonates (JAs) together with jasmonic acid and its offshoots are lipid-derived endogenous hormones that play key roles in both developmental processes and different defense responses in plants. JAs have been studied intensively in the past decades for their substantial roles in plant defense comebacks against diverse environmental stresses among model plants. However, the role of this phytohormone has been poorly investigated in the monocotyledonous species against abiotic stresses. In this study, a JA biosynthesis mutant *opr7opr8* was used for the investigation of JA roles in the salt stress responses of maize seedlings, whose roots were exposed to 0 to 300 mM NaCl. Foliar stomatal observation showed that *opr7opr8* had a larger stomatal aperture than wild type (WT) (B73) under salinity stress, indicating that JA positively regulates guard cell movement under salt stress. The results regarding chlorophyll content and leaf senescence showed that *opr7opr8* exhibited delayed leaf senescence under salt stress as compared to WT, indicating that JA plays a role in salt-inducing cell death and subsequent leaf senescence. Moreover, the morphological parameters, including the length of the shoots and roots, and the fresh and dry weights of the shoots and roots, showed that after 7 days of salt treatment, *opr7opr8* had heavier and longer shoots than WT but slighter and shorter roots than WT. In addition, ion analysis showed that *opr7opr8* accumulated less sodium but more potassium in the leaves than WT but more sodium and less potassium in the roots than WT, suggesting that JA deficiency causes higher salt stress to the roots but less stress to the leaves of the seedlings. Reactive oxygen species (ROS) analysis showed that *opr7opr8* produced less H_2_O_2_ than WT in the leaves but more H_2_O_2_ in the roots under salt treatment, and correspondingly, ROS-scavenging enzymes superoxide dismutase (SOD), catalase (CAT), and ascorbate peroxidase (APX) showed a similar variation, i.e., *opr7opr8* has lower enzymatic activities in the shoots but higher activities in the roots than WT under salt treatment. For osmotic adjustment, *opr7opr8* produced less proline in the shoots at 100 and 300 mM NaCl treatments but more in the roots than the WT roots under all salt treatments. In addition, the gene expression for abscisic acid (ABA) biosynthesis under salt stress was investigated. Results showed that the expression levels of four key enzymes of ABA biosynthesis, *ZEP1*, *NCED5*, *AO1*, and *VP10*, were significantly downregulated in the shoots as compared to WT under salt treatment. Putting all the data together, we concluded that JA-deficiency in maize seedlings reduced the salt-stress responses in the shoots but exaggerated the responses in the roots. In addition, endogenous JA acted as a positive regulator for the transportation of sodium ions from the roots to the shoots because the mutant *opr7opr8* had a higher level of sodium in the roots but a significantly lower level in the shoots than WT. Furthermore, JA may act as a positive regulator for ABA biosynthesis in the leaves under salt stress.

## 1. Introduction

Salt stress is one of the most serious abiotic stresses restraining the production of agricultural crops worldwide, specifically in arid and semi-arid areas. Grounded on the FAO/UNESCO report, 397 million hectares (approximately 3.1% of the world’s total land area) is affected by salt stress [1]. In addition, land degradation due to soil salinization has become a major global issue for maintainable agriculture in arid and semi-arid regions. Salt stress affects almost all aspects of plant growth and development including seed germination and the vegetative and reproductive growth development of plants [2]. A high salinity causes ionic toxicity, macro and micro nutrient (Na, K, P, Ca, Fe, Zn, etc.) deficiencies, and limits water uptake from the soil, thus reducing photosynthesis and metabolic processes under oxidative stress [2]. To deal with the biotic and abiotic stresses, a number of plants have developed multifaceted mechanisms to survive in adverse conditions including the saline soils. Salt tolerance either by salt elimination or accumulation within the cells is an economic trait for crops that helps them to produce a relatively high yield under saline soil conditions.

Phytohormones like abscisic acid (ABA), gibberellins (GA), ethylene (ET), salicylic acid (SA), jasmonates (JA), auxins (IAA), cytokinins (CK), brassinosteroids (BR), and strigolactones (SL) play positive roles in improving the tolerance of crops against abiotic stresses [3]. Some of them, such as abscisic acid, have been identified as stress hormones. Classically, abscisic acid is a hormone responsible against abiotic stresses such as drought, salt, cold, heat, and high-temperature stresses [4]. ABA upregulates the turgor pressure in cells, synthesizes osmoprotectants, and regulates the activity of antioxidants conferring dehydration tolerance. ABA activates the expression of a number of responsive genes including the genes of late embryogenesis abundant (LEA) proteins, dehydrins (DHNs), and other defensive proteins that play a fundamental protective role for membranes, organelles, and metabolic processes during water limitation [5]. Moreover, ABA closely interacts with several other stress-response hormones including SA, ET, and JA during the protective response to abiotic stresses [6]. Interestingly, evidence is increasing that growth-promoting hormones including IAA, GA, and CK play an integral part in plant responses to heat, salt, cold, and other stresses [7,8,9]. In general, ABA is regarded as the universal stress hormone. However, the hormonal crosstalk of ABA with other hormones is crucial to fine-tune plant defense responses against abiotic stresses or combinations of abiotic with biotic stresses.

Jasmonic acid (JA) and its offshoots, such as methyl jasmonate (MeJA), jasmonoyl-L-isoleucine (JA-Ile), and jasmonoyl-L-Tryptophan (JA-Trp), are collectively stated as jasmonates (JAs). These are fatty-acid-derived cyclopentanone compounds that occur ubiquitously and entirely in the plant kingdom [10,11] and serve as natural growth regulators in plant species [12]. These compounds play crucial roles in many plant biological processes, i.e., seed maturing, reproductive development, leaf senescence, root development, trichome and tendril formation, and the biosynthesis of many secondary metabolites in response to environmental stresses [13,14]. In model plants tomato and Arabidopsis, jasmonic acid has been deeply studied for their defensive role against insect and pest attacks. JA mutants, such as *fad3/7/8* [15], *opr3/dde1* [16,17], *aos/dde2* [18], and *coronatine insensitive1* (*coi1*) [19], are all male sterile, suggesting that JA is required for anther/pollen development in plants. All the above mutants except *opr3* have been shown to be susceptible to necrotrophic pathogens and insect pests, indicating that JA is an important element for plant responses to biotic stresses [20]. Interestingly, other JA signaling mutants, such as *jar1* [21] and *jin1/myc2* [22], are fertile but still susceptible to pathogens [22]. Similar results for defense responses have been obtained in tomato. In tomato, the systemin perception mutant *spr1* [23], JA biosynthesis mutant *spr2* [24], and JA perception mutant *spr6/jai1* [25] are impaired in the expression of wound-induced proteinase inhibitors (PIs) and are susceptible to insects and pests [24]. In addition to biotic stress, a number of studies have shown that JAs have taken significant protective responses against abiotic stresses including heavy metals [26], salt [27,28,29], drought [30], heat [31], and cold stress [32]. Salinity is indisputably a foremost abiotic stress factor that limits crop production by initiating ionic and osmotic stresses [33]. For salinity, JAs have been intensively studied as the positive regulators of salt tolerance [29,34]. For example, the foliar spray of MeJA can effectually lessen salt toxicity symptoms in soybean seedlings [35]. In grapevine, the foliar application of jasmonic acid can save plant growth in the salt-sensitive cell lines [34]. The exogenous application of JAs under saline stress improved the performance of safflower by a collective increase in chlorophyll a, b, photosystem II (Fv/Fm), leaf area index (LAI) [36]. In addition, the foliar application of JA to the seedlings of strawberries regulated enzymatic and non-enzymatic antioxidant activities, reduced lipid peroxidation, and increased the potassium content under salt stress [37]. The foliar spray of JA to the soybean seedlings enhanced the soluble protein content, antioxidant enzyme activity, and membrane stability index of the leaves [38]. In common wheat (*Triticum aestivum*), the *TaAOC1* gene encodes an allene oxide cyclase (AOC) enzyme of the JA biosynthesis pathway, and the over-expression of *TaAOC1* in Arabidopsis elevates jasmonic acid levels and promotes saline tolerance, suggesting that jasmonic acid positively regulates the salt tolerance in wheat [35]. However, there are also several reports available that suggest a negative role of JAs for the salt tolerance of plants. For example, rice mutants *cpm2* and *hebiba* are impaired in the function of allene oxide cyclase (AOC) of their JA biosynthesis pathway. These mutants were resistant to salt and drought stress. Interestingly, both mutants showed better scavenging of reactive oxygen species (ROS) under stress conditions [39]. In wild soybean (*Glycine soja*), the expression level of the GsJAZ2 gene was induced by varied abiotic stresses, and over-expression of GsJAZ2 in Arabidopsis enhanced its tolerance to saline stress [40]. Maize is an important crop for global food security and its production is restricted by environmental stresses especially by drought and soil salinity. Information about maize plant tolerance to salt stress is very limited so far. In this study, the molecular bases of the jasmonate-regulating salt tolerance of maize plants were investigated using the JA-deficient mutant *opr7opr8*. We found that JA-deficiency in maize seedlings reduced the salt-stress responses in the shoots but exaggerated the responses in the roots because JA takes an essential role in Na^+^ transportation from the root to shoots and JA positively regulates ABA biosynthesis in the leaves under salt stress.

## 2. Results

### 2.1. Jasmonate Is a Required Signal for Stomata Closure under Salt Stress

**Figure 1:**
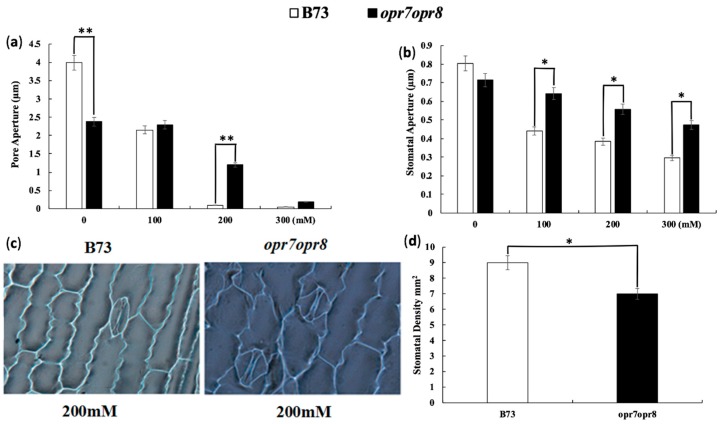
*opr7opr8* displays a higher stomatal aperture than wild type (B73) under salt (NaCl) treatment. (**a**) Pore aperture index of stomata. (**b**) Stomatal aperture index (SAI) of B73 and *opr7opr8* after 24 h salt treatment with 0 to 300 mM NaCl. (**c**) Stomata image of B73 and *opr7opr8* after 24 h under 200 mM NaCl treatment at 40x magnification. (**d**) Stomatal density on the third leaf of B73 and *opr7opr8*. The asterisks denote significant differences between B73 and the *opr7opr8* mutant at *p* < 0.05 (*) or *p* < 0.01 (**) by analysis of variance.

Stomata closure is the early response of plants to water stresses. Stomata closure largely reduces water loss by transpiration during the water stress period. In this study, we noted that JA-deficient mutant *opr7opr8* [41] has delayed “wilting,” a water loss symptom of plants under drought and salt-stresses, in comparison to wild type (WT), indicating that the stomata response to water stress of *opr7opr8* could be different from that of WT. We counted the stomatal density in the leaves of *opr7opr8* and WT, and the results showed that *opr7opr8* has a lower stomatal density in their leaves than WT after salt stress (Figure 1d), indicating that endogenous JA positively regulates stomata formation during leaf development. Stomatal visualization under salt stress at 0, 100, 200, and 300 mM salt stress showed that *opr7opr8* has fewer stomata in their leaves than wild type (WT), implying that *opr7opr8* may lose water less during water stress than WT. Stomatal closure during salt stress was studied. The results showed that after 24 h of salt treatment, the pore aperture index (PAI) and stomatal aperture index (SAI) of *opr7opr8* were higher than those of WT under salt stress treatments (100, 200, and 300 mM) (Figure 1a,b), indicating that the JA deficiency in *opr7opr8* slows down the stomatal closure during salt stress in comparison with WT. Figure 1c shows that the stomatal opening in *opr7opr8* is wider than in B73 after 24 h of 200 mM NaCl application. The stomatal visualization of leaves under 0, 100, and 300 mM NaCl treatments is shown in Figure S1. At later hours of salt treatment, *opr7opr8* and WT keep their stomata close, and there is water loss through the epidermal cells in both genotypes.

### 2.2. opr7opr8 Mutant Showed Delayed Leaf Senescence under Salt Stress

**Figure 2:**
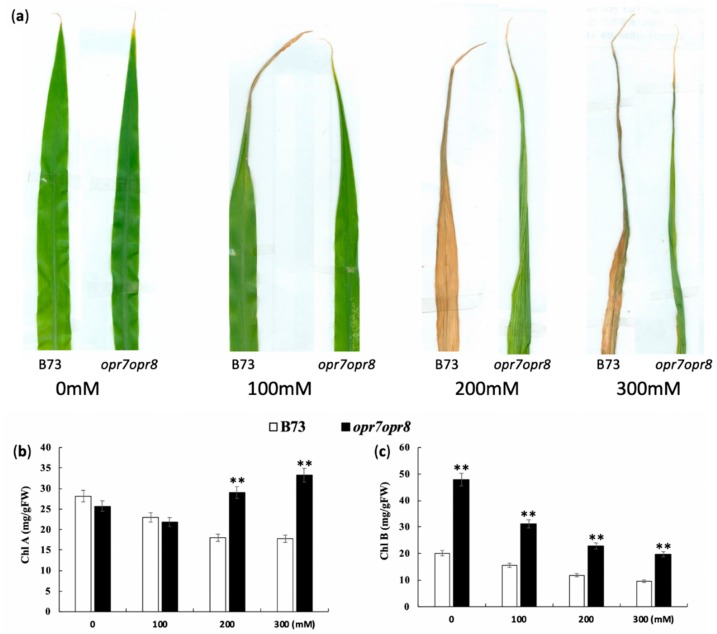
*opr7opr8* undergoes delayed leaf senescence upon salt (NaCl) stress compared to B73. (**a**) Chlorosis symptom of leaves of B73 and *opr7opr8* seedlings whose roots were treated with 0–300 mM NaCl in the hydroponic system. The third leaves of the two genotypes were used to take the pictures at 7 days of salt treatment. (**b**) Measurement of chlorophyll A content in the leaves of B73 and *opr7opr8* seedlings at 2 days of salt stress. (**c**) Measurement of chlorophyll B content in the leaves of B73 and *opr7opr8* seedlings at 2 days of salt stress. The asterisks denote significant differences between wild type (WT) and *opr7opr8* at *p* < 0.01 (**) by analysis of variance.

To dissect JA roles in leaf senescence under salt stress, the seedlings of *opr7opr8* and WT (at V3-stage) were applied with different concentrations of NaCl (0, 100, 200, and 300 mM) in a hydroponic system. Data were collected for different physiological parameters, i.e., fresh root length (FRL), fresh shoot length (FSL), fresh root weight (FRW), fresh shoot weight (FSW), dry root weight (DRW), and dry shoot weight (DSW), in these salt treatment experiments. We observed that the leaves of *opr7opr8* were less wilted and necrotic as compared to B73 under salt stress. At treatment of 100 mM sodium chloride, the tips of the leaves of *opr7opr8* and B73 were becoming yellowish after 7 days of salt treatment (Figure 2a), but no substantial differences in leaf senescence were experienced among the two genotypes under 100 mM sodium chloride treatment. The treatment of 200 mM sodium chloride strongly activated leaf senescence of B73 and *opr7opr8*, but the symptoms of leaf rolling and necrosis in *opr7opr8* developed slower than those in B73. At 7 days of treatment, all the leaves of B73 were dried and yellow, but the leaves of *opr7opr8* were rolled and green (Figure 2a). At treatment of 300 mM sodium chloride, the symptoms of leaf rolling, yellowing, and drying were similar to the treatment of 200 mM sodium chloride, but the symptoms under 300 mM sodium chloride developed 2 days earlier than those under 200 mM sodium chloride. At 5 days of 300 mM sodium chloride treatment, all seedlings of B73 were dried, but the new leaves (third and fourth leaf) of *opr7opr8* seedlings remained green (Figure 2a). We measured the chlorophyll contents of the shoots of B73 and *opr7opr8* at 2 days of salt treatment (Figure 2b,c). The result showed that *opr7opr8* had a significantly higher chlorophyll A content than B73 at 200 and 300 mM sodium chloride treatments and significantly higher chlorophyll B at all the salt concentrations including 0 mM. All the results indicated that JA-deficient mutant *opr7opr8* underwent delayed leaf senescence upon salt stress as compared to WT, suggesting that endogenous JA acts as a negative regulator for salt stress response in maize.

### 2.3. opr7op8 Displayed Better Growth in the Shoots but Worse Growth in the Roots Than B73 under Salt Stress

**Figure 3:**
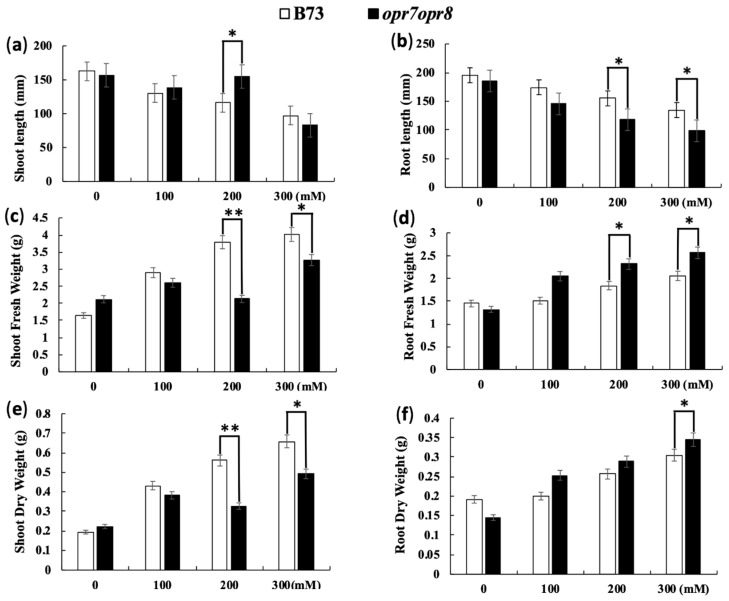
Salt treatments inhibit the growth of shoots and roots of B73 and *opr7opr8* seedlings. The V3-stage plants of B73 and *opr7opr8* were treated with 0–300 mM NaCl in the hydroponic system, and the (**a**) shoot length, (**b**) root length, (**c**) shoot fresh weight, (**d**) root fresh weight, (**e**) shoot dry weight, and (**f**) root dry weight were measured after 7 days of salt treatments. The shoot length is the distance from the first node (the coleoptiles node) to the tip of the leaves. The root length was measured from the first node to the far-end of the root system. The asterisks show significant differences for WT and the mutant at *p* ≤ 0.05 (*) or *p* ≤ 0.01 (**) by analysis of variance.

In this study, we investigated the growth inhibition difference under salt stress between *opr7opr8* and WT. The shoot and root length of *opr7opr8* and WT showed growth inhibition under salt treatments (Figure 3a,b). As the salt concentration increased, the effects of growth inhibition were exaggerated. However, we observed that the shoot length of *opr7opr8* was longer than that of WT after 7 days of 100 and 200 mM salt treatments, but the root length of *opr7opr8* was significantly shorter than that of WT. With the concentration of NaCl increasing, the root length of both genotypes decreased, but this decrease was sharper in *opr7opr8* than in B73. Our result indicates that the growth inhibition to *opr7opr8* shoots was slighter than that of B73, but to the roots, it was stronger than that of B73 (Figure 3a,b), suggesting that to JA-deficient mutant *opr7opr8*, salt treatments cause stronger damage to the roots but slighter damage to the leaves as compared to B73. The *opr7opr8* plants showed less shoot fresh weight and shoot dry weight under salt stress as compared to B73 (Figure 3c,e). However, for the roots, *opr7opr8* showed a higher shoot fresh weight and shoot dry weight in comparison with WT (Figure 3d,f). For WT plants, as the salt concentration increased, the shoot fresh and dry weights and root fresh and dry weights increased (Figure 3c–f).

### 2.4. opr7opr8 Accumulated Less Sodium in the Leaves but More Sodium in the Roots Than WT under Salt Stress

**Figure 4:**
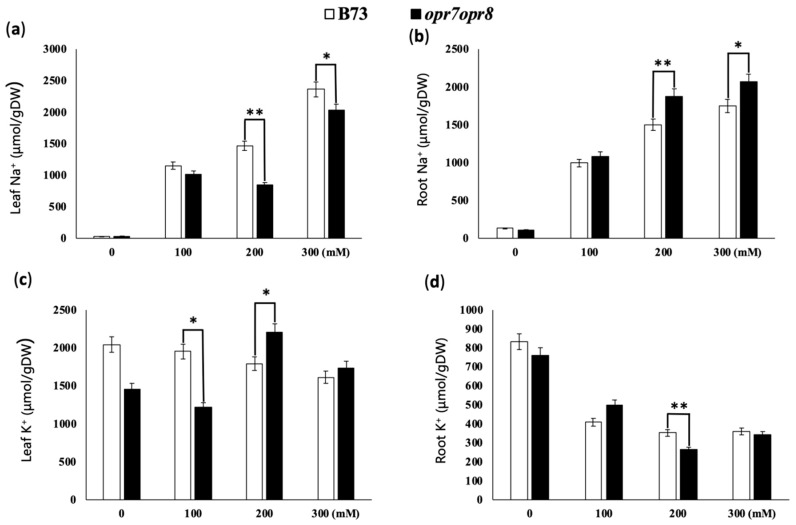
The leaves and roots of B73 and *opr7opr8* seedlings accumulate sodium and potassium under NaCl treatments. The samples were taken at 7 days after salt treatment. (**a**) Na^+^ ion content in the leaf of B73 and *opr7opr8* plants. (**b**) Na^+^ ion content in the roots. (**c**) K^+^ ion content in the leaves. (**d**) K^+^ ion content in the roots. The asterisks denote significant differences between WT and the mutant at *p* ≤ 0.05 (*) or *p* ≤ 0.01 (**) by analysis of variance.

In this study, we analyzed sodium and potassium accumulation in the roots and leaves of B73 and *opr7opr8* plants treated at 0, 100, 200, and 300 mM NaCl. Our results showed that the higher the concentration of NaCl applied, the higher the content of Na^+^ in the leaves and roots of B73 and *opr7opr8* plants detected (Figure 4a,c). However, B73 and *opr7opr8* had different levels of salt content in the leaves and roots. In the leaves, *opr7opr8* accumulated significantly fewer Na^+^ than WT (Figure 4a), indicating that in *opr7opr8,* the Na^+^ transportation from the roots to the leaves was reduced during the salt stress. In the roots, *opr7opr8* had a higher Na^+^ content than WT (Figure 4c), indicating that the mutant was compromised to exclude Na^+^ out of the roots or to transport Na^+^ from the roots to the shoot. Homeostasis of potassium ions and sodium ions plays a crucial role in plant development under salt stress conditions. A reduced K^+^/Na^+^ ratio is the most commonly observed physiological feature of plants challenged by salt stress [42]. In this study, we observed that the K^+^ content declined in the roots and leaves of both genotypes with the increase in salt solution concentration (Figure 4b,d). Both genotypes showed different K^+^ contents in the shoots and roots under salt stress. At the level of 100 mM NaCl, K^+^ contents in the shoots and roots in WT were higher than those in the mutant. At the level of 200 and 300 mM NaCl, *opr7opr8* retained higher K^+^ contents in the shoot than WT, but in the roots, *opr7opr8* accumulated lower contents than WT. Putting the sodium and potassium results together, we concluded that the *opr7opr8* mutant retained more sodium in the roots but less sodium in the leaves under salt stress, and that for potassium was the opposite to sodium, suggesting that JA plays an important role in maize plants regarding Na^+^ ion uptake and transportation under the salinity condition. Salinity may cause other mineral nutrient deficiencies or imbalances due to the accumulation of Na^+^ ions in the roots and shoots.

### 2.5. opr7opr8 and WT Accumulated a Different Level of ROS under Salt Stress

**Figure 5:**
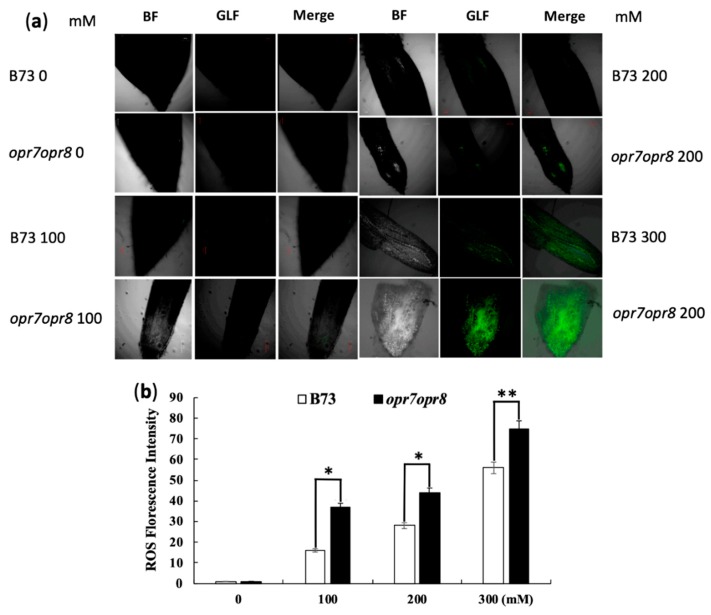
Reactive oxygen species (ROS) production in the meristematic zone of the roots of B73 and *opr7opr8* under 0–300 mM NaCl treatments. (**a**) Visualization detection of ROS production under bright field (BF) and green light (480–550 nm) fluorescence (GLF) with a confocal microscope using 2′,7′-dichlorofluorescin diacetate (H2DCFDA) at 4 h after salt treatments. (**b**) Relative fluorescence quantification of ROS production in root meristematic zones of B73 and *opr7opr8* under salt treatments by software ImageJ scan. The values of B73 and *opr7opr8* were given 1 at 0 mM for relative ROS quantification. The asterisks denote significant differences between WT and the mutant at *p* ≤ 0.05 (*) or *p* ≤ 0.01 (**) by analysis of variance.

To investigate the JA roles involved in the ROS production under salt stress, a ROS visualization experiment was performed in plant roots by using 2′,7′-dichlorofluorescin diacetate (H2DCFDA). Root samples were collected 4 h after salt stress and immediately underwent ROS detection treatment. The meristematic zone of the root tips of nine individual plants for each treatment was used as the samples. ROS production in the meristematic zone of the roots was detected under a confocal microscope for 0, 100, 200, and 300 mM NaCl treatments (Figure 5a). The relative fluorescence quantification of ROS production in the root meristematic zone is shown in Figure 5b. Our results show that the relative fluorescence value of ROS in *opr7opr8* roots was significantly higher than in WT (Figure 5a,b) at 200 and 300 mM salt treatments, suggesting that the roots of *opr7opr8* are highly sensitive to salt damage under salt stress.

**Figure 6:**
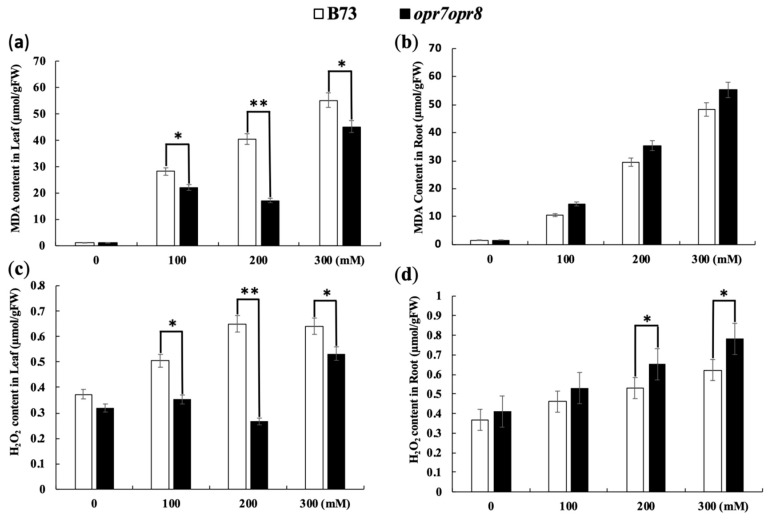
Malondialdehyde (MDA) and H_2_O_2_ accumulation in *opr7opr8* and B73 under salt stress. The MDA levels in the (**a**) leaves and (**b**) roots were detected after 48 h of 0–300 mM NaCl treatments. The H_2_O_2_ content in the (**c**) leaves and (**d**) roots was measured after 48 h of salt treatments. The asterisks denote significant differences between WT and the mutant at *p* ≤ 0.05 (*) or *p* ≤ 0.01 (**) by analysis of variance.

In this study, the H_2_O_2_ level was also determined in the roots and leaves of both genotypes under salt treatments. H_2_O_2_ causes plant cell death as a result of environmental stresses, so its regulation is crucial in the growth and developmental events [43]. In the leaves and roots of B73 and *opr7opr8*, the production of H_2_O_2_ increased with the increase in salt concentration (Figure 6c,d). H_2_O_2_ production was significantly higher in the leaves of B73 at 200 mM NaCl treatment, whereas there was no significant increase in H_2_O_2_ production among the leaves of *opr7opr8*. Our study showed that *opr7opr8* produced significantly more H_2_O_2_ in its roots as compared to its leaves. Salt treatments increased H_2_O_2_ levels in the roots and leaves of both genotypes (Figure 6c,d), and the genotypes had significantly different H_2_O_2_ levels. In the roots, *opr7opr8* showed a higher H_2_O_2_ level than WT, and in the leaves, *opr7opr8* produced a lower H_2_O_2_ level than WT.

Oxygen-free radicals cause lipid peroxidation in an organism. An upsurge in free radicals under stress conditions causes overproduction of malondialdehyde (MDA). The MDA level is known as a marker of oxidation stress and the antioxidant activity in plants under stress conditions. In this study, MDA contents were detected in both genotypes under the salt treatments. The salt treatments resulted in MDA accumulation in B73 and *opr7opr8*. The MDA levels increased in the leaves and roots of both genotypes with the increase in salt concentration (Figure 6a,b). In the roots, the MDA accumulation in *opr7opr8* was significantly higher than that in WT (Figure 6b), whereas in the leaves, the MDA level in *opr7opr8* was significantly lower than that in WT, indicating that *opr7opr8* suffered from a stronger lipid peroxidation in the roots than WT but slighter lipid peroxidation in the leaves than WT under salt stress.

### 2.6. opr7opr8 Displayed Different Antioxidant Enzyme Activities from WT under Salt Treatment

**Figure 7:**
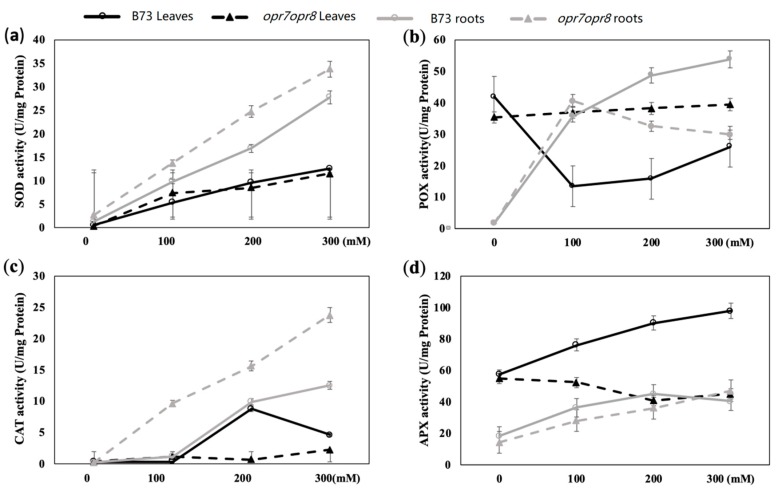
ROS-scavenging enzyme activities in the shoots and roots of *opr7opr8* and B73 seedlings under 0–300 mM NaCl treatments. The samples were taken 2 days after the salt was applied to the hydroponic solution. (**a**) SOD activity in leaves and roots, (**b**) POX activity in leaves and roots, (**c**) CAT activity in leaves and roots, and (**d**) APX activity in leaves and roots.

The enzymatic antioxidant defense system including superoxide dismutase (SOD), peroxidase (POD), catalase (CAT), ascorbate peroxidase (APX), and glutathione peroxidase (GPX) is important for the plant to cope with ROS bursts during salt stress. In this study, the enzymes such as SOD, POD, CAT, and APX were analyzed in the roots and leaves of *opr7opr8* and B73 plants under salt treatments. The activity of SOD increased with the increase in salt concentration in *opr7opr8* and B73 plants. The SOD activity in the leaves of *opr7opr8* was lower than that of WT under 200 and 300 mM NaCl treatments (Figure 7a), but in the roots, the SOD activity of *opr7opr8* was higher than that of WT under 100, 200, and 300 mM NaCl treatments (Figure 7a). For peroxidase (POX) (total class III peroxidases), salt treatments activated POX activity in the roots of B73 and *opr7opr8*. In the roots of B73, the POX activity increased with the increase in salt concentration (Figure 7b). In the roots of *opr7opr8*, the POX activity at 100 mM NaCl treatment was higher than 200 and 300 mM NaCl. *opr7opr8* roots showed a higher POX activity than WT at 200 and 300 mM NaCl treatment. In the leaves, the POX activity was downregulated in WT under salt treatments, but for *opr7opr8* salt, the treatments did not affect the POX activity in the leaves (Figure 7b). For CAT, the fluctuation in enzyme activity under salt treatment was similar to SOD. The salt treatments increased the CAT activity in the roots of B73 and *opr7opr8*, and the mutant had a higher activity than WT (Figure 7c). In the leaves, salt treatments increased the CAT activity in B73 but decreased in *opr7opr8* (Figure 7c). The mutant exhibited a significantly lower level of CAT activity in the leaves than in WT. Ascorbate peroxidase (APX) is a major ROS-scavenging enzyme controlling intracellular ROS levels in varied stresses. The variation in APX activity under salt treatments was similar to the POX activity. In the roots, the APX activity in B73 and *opr7opr8* increased with the increased salt concentration (Figure 7d). At 100 and 200 mM NaCl treatments, in the roots of *opr7opr8,* the APX activity was lower than that of WT. In the leaves, the APX activity in B73 was elevated when the salt concentration increased, but, in the leaves of *opr7opr8*, the APX activity had no significant change under salt stress (Figure 7d). In the leaves, *opr7opr8* was significantly lower than that of WT for APX activity under salt stress. Putting the four enzyme results together, we saw that *opr7opr8* had a significantly different level of ROS-scavenging enzyme activity in the roots and shoots under the salt treatments as compared to WT, indicating that the JA deficiency in *opr7opr8* plants caused ROS-scavenging enzyme genes to be differentially expressed under salt stress.

### 2.7. opr7opr8 Exhibited Different Levels of Glutathione Reductase (GR) and Glutathione-S-Transferase (GST) Activities from WT under Salt Treatment

**Figure 8:**
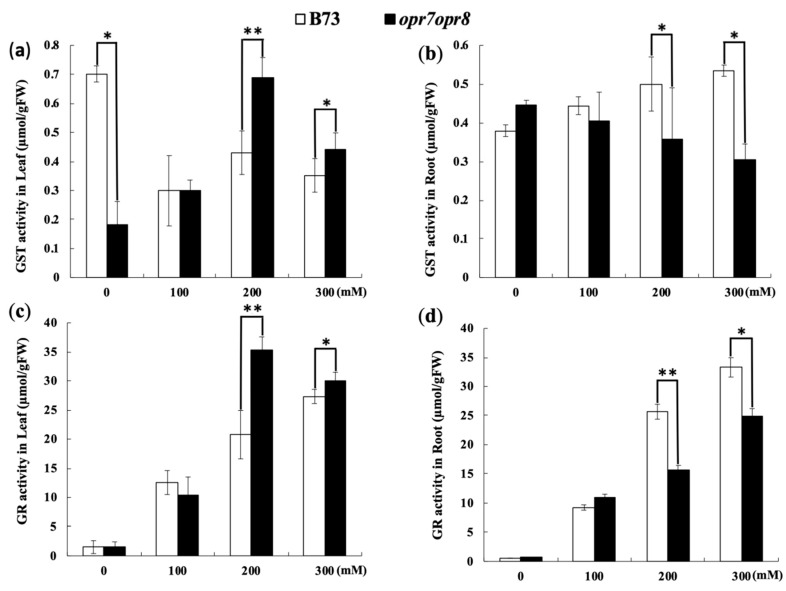
Enzymatic activities of glutathione reductase (GR) and glutathione-S-transferase (GST) in B73 and *opr7opr8* seedlings under 0–300 mM NaCl treatments. The samples were taken 2 days after the salt treatments. GST activity in (**a**) the leaves and (**b**) roots and GR activity in the (**c**) leaves and (**d**) roots. The asterisks denote significant differences between WT and the mutant at *p* ≤ 0.05 (*) or *p* ≤ 0.01 (**) by analysis of variance.

Except for SOD, POD, CAT, and APX, glutathione reductase (GR) and glutathione-S-transferase (GST) can also act as the enzymatic antioxidants in plants under abiotic stresses. In this study, the GR and GST activities were analyzed in *opr7op8* and B73 plants under salt treatments. The GR activity increased with the increase in salt stress in the leaves and roots of *opr7op8* and B73 plants (Figure 8c,d). In the roots, the GR activity in *opr7opr8* was higher than that in B73, but in the leaves, *opr7opr8* was lower than WT at salt treatments of 200 and 300 mM NaCl (Figure 8c). For GST activity, in the leaves, salt treatments reduced the GST activity in B73 but activated GST activity in *opr7opr8* (Figure 8a). At 200 and 300 mM NaCl, the GST activity in the leaves of *opr7opr8* was higher than that in WT. In the roots, salt treatments slightly induced the GST activity in WT but slightly inhibited in *opr7opr8* (Figure 8b). At 200 and 300 mM NaCl, WT was significantly higher than *opr7opr8* for GR activity in the roots (Figure 8d).

### 2.8. opr7opr8 Accumulated More Proline in the Roots but Less in the Leaves Than WT under Salt Stress

**Figure 9:**
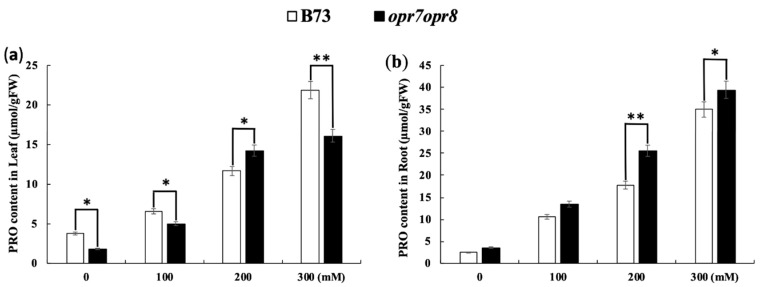
Proline contents were detected in (**a**) the leaves and (**b**) roots at two days after 0–300 mM NaCl was applied to the roots. The asterisks denote significant differences between WT and the mutant at *p* ≤ 0.05 (*) or *p* ≤ 0.01 (**) by analysis of variance.

Under abiotic stresses, plants tend to accumulate soluble osmotic adjustment substances such as proline to protect the cellular structure and enzyme activity against osmotic and ionic stresses. In this study, the proline content accumulation was tested in the leaves and roots of *opr7opr8* and B73 plants treated with 0, 100, 200, and 300 mM NaCl. The results showed that proline production was highly induced by salt treatments in the leaves and roots in both genotypes (Figure 9a,b). In the leaves, the proline accumulation level in *opr7opr8* was significantly lower than that in WT, except under 200 mM NaCl (Figure 9a). In the roots, *opr7opr8* accumulated a higher proline level than WT (Figure 9b). Our results suggest that JA is involved in osmotic regulation under salt stress.

### 2.9. Endogenous JA Production Is Required for Transcriptional Activation of ABA Biosynthesis Genes under Salt Stress

**Figure 10:**
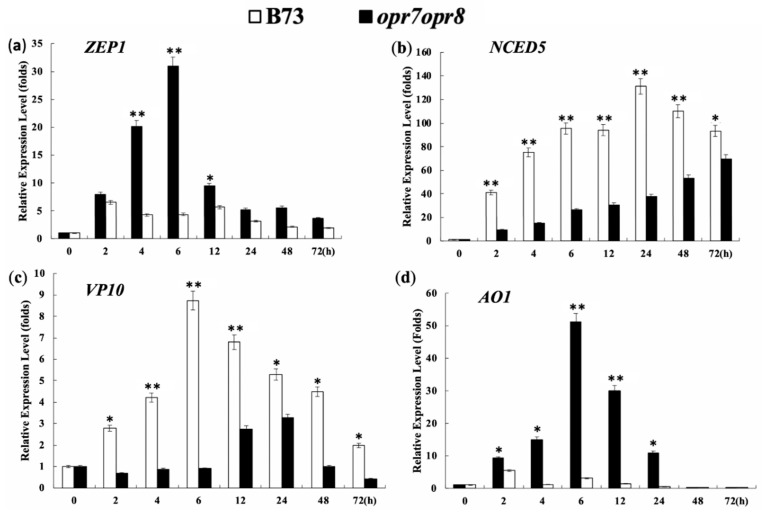
Quantitative real-time polymerase chain reaction (qRT-PCR) expression level analysis of (**a**) *NCED5*, (**b**) *AO1*, (**c**) *VP10*, and (**d**) *ZEP1* genes in the leaves of the B73 and *opr7opr8* plants under 200 mM NaCl treatment. The relative expression level was calculated according to the expression of the gene at 0 h of treatment. The asterisks denote significant differences between WT and the mutant *opr7opr8* at *p* ≤ 0.05 (*) or *p* ≤ 0.01 (**) by analysis of variance.

Abscisic acid (ABA) plays an important role for plants to deal with abiotic stresses, including drought and soil salinity. In this study, we tested the expression levels of four key genes, *ZEP1*, *NCED5, VP10*, and *AO1*, of the ABA biosynthesis pathway under salt treatment in the leaves and the roots of *opr7opr8* and WT. ZEP1 (zeaxanthin epoxidase1) is the initial enzyme of the ABA biosynthesis pathway in maize. NCED (9-cisepoxycarotenoid dioxygenase) catalyzes the oxidative cleavage of epoxy-carotenoid 9-cis-neoxanthin, the first step of abscisic-acid biosynthesis from carotenoids [44]. *Vp10* (*viviparous10*) encodes the ortholog of Cnx1, which catalyzes the final common step of molybdenum cofactor (MoCo) synthesis. The sulfur-containing form of MoCo, MoCo-S, is a required cofactor of AO1 activity [45]. AO1 (aldehyde oxidase 1), a molybdenum-containing oxidoreductase, catalyzes the final step of ABA biosynthesis, the conversion of abscisic aldehyde to ABA. In our experiments, the expression of all the four genes showed a similar induction pattern, that is, in the leaves of B73, the four genes were highly induced by salt treatment, but not induced or slightly induced in the *opr7opr8* mutant (Figure 10a,c–e). Our results showed that 200 mM NaCl strongly induced the *ZEP1* expression in WT, and the maximum induction was more than 30 times at 6 h of treatment in comparison with that at 0 h (Figure 10d). However, in the mutant *opr7opr8,* the *ZEP1* gene was just slightly induced at the early time points (2 to 12 h of the treatment) and the maximum induction was about 5 times at 2 h of treatment (Figure 10d). Overall, the *ZEP1* gene expression in *opr7opr8* was significantly lower than that of WT at 4 to 72 h of salt treatment (Figure 10d). At 200 mM salt stress, the *NCED5* gene was highly upregulated in the leaves of B73, as compared to mutant *opr7opr8* (Figure 10a). At 24 h of 200 mM salt treatment, the expression level of the *NCED5* gene in B73 was increased to 130 times the expression level at 0 h. Then, it decreased at longer time points. While in the mutant, it upregulated gradually from 2 to 72 h salt stress, the expression level was highest at 72 h (Figure 10a). The *AO1* gene was also highly induced by salt treatment in WT but not in the mutant *opr7opr8* (Figure 10c). The expression level of the *AO1* gene was increased to 50 times at 6 h of salt treatment compared to 0 h of treatment (Figure 10c). In *opr7opr8*, the maximum induction was less than 5 times, which happened at 2 h of salt treatment (Figure 10b). At the timepoints of 2 to 24 h of the treatment, the *AO1* expression level in *opr7opr8* was significantly lower than that in WT (Figure 10c). Salt treatment induced the expression of the *VP10* gene in WT and the mutant (Figure 10e). For WT, the induction peak appeared at 6 h of salt treatment, and for the mutant, it appeared at 24 h (Figure 10e), indicating that *VP10* induction by salt treatment was faster in WT than in the mutant. For all time points, the induction level of *VP10* expression in WT was significantly higher than in the mutant. Putting the data of the four genes together, we saw that four key genes of the ABA biosynthesis pathway, *NCED5*, *ZEP1*, *AO1*, and *VP10*, were strongly induced in the WT plants by salt stress but were significantly less expressed in the mutant with no or a slight induction by salt treatment, indicating that endogenous JA is a positive factor for ABA accumulation under salt stress in maize.

## 3. Discussion

Salinity is one of the most ubiquitous environmental stresses limiting the yield of agricultural crops with adverse effects on the vegetative and reproductive growth of plants [46]. Growing on saline-alkali soil, the plant has to tolerate the root-absorbed excessive sodium ions that have a damaging effect on biochemical reactions and causes ionic, osmotic, and oxidative stresses to plant cells [46,47]. Phytohormones have long been considered essential endogenous molecules regulating plant development and tolerance to diverse environmental stresses including salinity stress [48]. ABA is well known as the endogenous signal molecules enabling plants to survive the severe adverse environmental conditions such as salt and drought stresses [49]. Increasing evidence supports the idea that jasmonic acid can play relevant functions in the abiotic stress response [50,51]. Up until now, three major lines of evidence have been reported for JA contribution in the adaptive response to salt stress. **(1)** Salt stress-activated JA biosynthesis in plants. Elevated JA levels were detected in a number of plant species such as Arabidopsis [52], tomato [53,54], rice [55,56], maize [57], and *Brassica rapa* [58] when challenged with salt stress, indicating that high levels of JAs accumulated in salt-challenged plants after salt treatment may function as a protection signal in plants against salinity stress. **(2)** The exogenous application of JA or MeJA to leaves or roots enhances the salt tolerance of plants. Here are several examples: The exogenous application of 30 μm JA applied 24 h after salt stress effectively reduced the sodium ion uptake in rice seedlings, especially in the salt-sensitive cultivars rather than the salt-tolerant cultivars [56]. A ten-micrometer solution of MeJA sprayed on rice varieties could effectively alleviate the symptoms of rice varieties to salinity stress [59]. An exogenous spray of 2 mM JA can enhance the tolerance of wheat seedlings to salt stress [28]. The foliar application of 20 to 30 µM MeJA can effectively lessen salinity stress symptoms and change endogenous ABA and GA_4_ levels in soybeans [37]. Foliar sprays of 1 mM SA and 0.5 mM JA stimulate the H^+^-ATPase activity of tonoplast and salt tolerance of soybean seedlings [60]. In grapevine, 10 to 50 µM JA treatments can save growth in the salt-sensitive cell lines, and the salt stress response of the lines is modulated by JA-signaling components such as JAZ proteins [34]. Exogenous sprays of 100 µM MeJA to the seedlings of *Brassica napus* mitigate the inhibitory effect of all salt treatments [61]. In maize, 10 µM JA applied to the seed before germination alleviates alkaline (Na_2_CO_3_) stress by improving the ascorbate glutathione cycle and glyoxalase system in the seedlings [62]. (**3)** Alterations of JA biosynthesis or interference of JA signaling affects the salinity tolerance of plants. A couple of JA biosynthesis or signaling mutants have been tested for their tolerance or susceptibility against abiotic stresses so far. The mutants of the JA biosynthesis enzyme AOC, *hebiba,* and *cpm2* in rice showed an increased salt tolerance [51]. Transgenic rice with overexpressed gene *CYP94*, encoding an inactivating JA-Ile catabolic enzyme, displayed enhanced salt tolerance [63]. The suppression of OsJAZ9, a repressor of JA signaling, produced a higher sensitivity to JA and increased sensitivity to salt [64]. The overexpression of OsJAZ8, a JA signaling suppressor, improved the salt tolerance of transgenic rice seedlings [65]. The results of the above four published works indicate that a JA-deficiency or JA signaling depression in transgenic rice causes improved salt tolerance. Putting all the three evidence lines together, we saw that the roles of exogenous JA applications are quite consistent from the different studies: The exogenous application of MeJA or JA can significantly enhance the tolerance of plants to salt stress. However, the roles of endogenous JA signals or JA signaling for salt tolerance can be varied, whose conclusion depends on the plant species. Obviously, more research works using JA biosynthesis or signaling mutants from different plant species are needed to clarify the endogenous JA roles involved in the adaptive response against abiotic stresses in plants.

In this study, *opr7opr8*, a maize JA biosynthesis mutant [41], was applied to identify the roles of endogenous JA in maize plants challenged by salt stress. Our results have shown that the shoots of *opr7opr8* display a number of symptoms weaker than those of WT under salt treatments, including delayed leaf senescence, less sodium accumulation, less ROS production, and less antioxidant enzyme activities in the leaves, among others. From these results, we can conclude that *opr7opr8* shoots are less sensitive to salt stress. Rice JA biosynthesis mutants *hebiba* and *cpm2* showed an increased salt tolerance [51]. Our research results indicated that JA biosynthesis mutant *opr7opr8* [43] resembles the rice JA biosynthesis mutants *hebiba* and *cpm2* [53] in the adaptive response against salt stress. Interestingly, in this study, we found that the roots of *opr7opr8* showed stronger symptoms such as more sodium accumulation and more ROS and antioxidant enzyme activities than WT under salt treatments, indicating that the roots of *opr7opr8* are more susceptible than WT. Comparing the data of shoots and roots, we suggested that endogenous JA differentially regulates salt responses in the shoots and roots in maize seedlings for acclimation to salinity in the soil.

Stomata are the vital organ of plants to control water loss under abiotic stresses. Stomatal closure can sharply reduce transpirational water loss under drought and salt stresses. It has been known that the exogenous application of methyl jasmonate or jasmonic acid elicits stomatal closure in a large number of plant species [66] including Arabidopsis [67], *Olea europaea* [68], and barley [69]. It has also been known that JA negatively regulates stomatal formation in Arabidopsis cotyledons [70]. However, the question of how endogenous JA is involved in stomatal closure under abiotic stresses remains unanswered. In this study, we observed that *opr7op8* had a larger stomatal aperture than WT (B73) under salinity stress (Figure 1), indicating that the JA-deficient mutant *opr7op8* is insensitive to stomatal closure under salt stress. This result implies that endogenous JA positively regulates guard cell movement for stomatal closure under water stress conditions.

A common symptom of damage by salinity stress is the growth inhibition, and leaf senescence will appear afterward during prolonged exposure to salt stress. In this study, we showed that after 7 days of the salt treatments, *opr7opr8* showed late leaf senescence as compared to WT (Figure 2), suggesting that endogenous JA in maize acts as a negative regulator for plant growth and leaf senescence under salt stress. We tested the fresh weight and dry weight and found that *opr7opr8* had a higher fresh/dry weight of shoots than WT, but a smaller fresh/dry weight of roots than WT. These results indicated that JA-deficiency in maize plants causes different stress strengths to the roots and shoots under salt treatments. In addition, we noted that both genotypes have a higher fresh/dry weight under higher NaCl concentrations (Figure 3c–f), suggesting that sodium and potassium accumulation in the leaves and roots could be the major reason of the biomass weight increase under salt stress. We tested the sodium and potassium contents in the shoots and roots, and the results showed that both genotypes have increasing sodium contents in the shoots and roots as the treatment salt concentration increases (Figure 4a,b). For example, under 300 mM NaCl at 7 days, WT leaves contained Na^+^ 3000 μmol/gDW (Figure 4a) and K^+^ 2000 μmol/gDW (Figure 4c), which is NaCl 0.176 g/gDW and KCl 0.151 g/gDW, indicating that the salts (NaCl and KCl) occupied 32.7% of the dry mass of the WT leaves. Similarly, *opr7opr8* leaves contained Na^+^ 2000 μmol/gDW (Figure 4a) and K^+^ 1500 μmol/gDW (Figure 4c), that is, salts (NaCl and KCl) occupied 23.0% of the dry mass. This calculation suggests that sodium and potassium accumulation in the leaves and roots promoted the increase in biomass weight under salt treatments.

Salinity induces the formation of reactive oxygen species (ROS) within plant cells, which is a well-known cause of damage to all components of the cell, including proteins, lipids, carbohydrates, and DNA [71]. To scavenge excessive levels of ROS, an effective system composed of non-enzymatic and enzymatic antioxidants is evolved in plants [72]. Non-enzymatic antioxidants include phenolic compounds, flavonoids, alkaloids, tocopherol, carotenoids, ascorbate (ASC), and glutathione (GSH) [73]. Enzymatic antioxidants include superoxide dismutase (SOD), peroxidase (POX), catalase (CAT), ASC peroxidase (APX), guaiacol peroxidase (GPX), glutathione reductase (GR), monodehydroascorbate reductase (MDHAR), and dehydroascorbate reductase (DHAR) [73,74]. A number of previous studies have shown that jasmonate mediates ROS production and antioxidant enzymatic activities under abiotic stresses [75]. An exogenous spray of 1 mM MeJA induced ROS accumulation and activated the activities of CAT, GPX, and APX in *Ricinus communis* leaves [76]. The exogenous application of 50 μM MeJA to sunflower (*Helianthus annuus* L.) elicited a fast increase in ROS content, followed by a marked increase in the activity of H_2_O_2_-scavenging enzymes such as GPX, APX, and CAT [77]. In grape, salt-tolerant cultivars have higher antioxidant enzyme activities [78]. In this study, we showed that JA-deficient mutant *opr7opr8* had a higher H_2_O_2_ level in the roots than WT under the salt treatments (Figure 6), indicating that endogenous JA may mediate ROS production or ROS-scavenging under abiotic stress. Meanwhile, we saw that *opr7opr8* had a lower H_2_O_2_ level in the shoots than WT. This result must indicate that the mutant *opr7opr8* suffered from milder salt stress than WT in the leaves because *opr7opr8* leaves have a significantly lower sodium content than WT (Figure 4). As for ROS-scavengers, *opr7opr8* had lower levels of SOD, CAT, and APX activities in the shoots than WT but higher activities in the roots than WT. This result indicates that JA-deficiency in maize seedlings causes strong ROS production and enzymatic activities of ROS-scavenging in the roots, but an opposite phenomenon in the shoots, suggesting that a differential mechanism of JA is involved in the salt response in the shoots and roots of the maize plant.

In many studies, ABA has been regarded as the most important phytohormone that confers abiotic stress tolerance in plants [79]. However, ABA crosstalk with other hormones is crucial to fine-tune plant responses to varied stresses. It is identified that the JA signaling pathway interacts with the ABA pathway via transcription factors such as MYC2, ABI5, and WRKY57 [80]. In our previous study, we reported that ABA production was dramatically reduced in the senescing leaves of *opr7opr8* compared to the wild type, indicating a significant role for JA in the regulation of ABA biosynthesis during leaf senescence in maize [41]. In this study, we quantified the transcriptional levels of four key genes (*NCED5*, *ZEP1*, *AO1,* and *VP10*) of the ABA biosynthesis pathway by quantitative polymerase chain reaction (PCR) in the mutant and WT. Our results exposed that the expression of these four genes was strongly activated by salt treatment (200 mM) in the leaves of WT but was just slightly induced or non-inducible in the mutant, indicating that *opr7opr8* was insensitive to salt stress for ABA biosynthesis activation.

In this study, we noted that the *opr7opr8* mutant under salt stress showed milder growth inhibition, a lower production of H_2_O_2_, lower level of ROS-scavenging enzymatic activities of SOD, CAT, and APX, lower production of MDA and proline, and lower expression of ABA biosynthesis genes in the leaves than WT. In the roots, *opr7opr8* under salt stress showed stronger growth inhibition and a much higher responsibility to salt. Our results indicated that endogenous JA in the maize plant might differentially regulate the adaptive response to salt stress in roots and shoots, suggesting that different JA-relevant mechanisms in roots and shoots might work in maize seedlings. Meanwhile, we observed that the *opr7opr8* mutant accumulated less sodium and more potassium in the shoots than WT but more sodium and less potassium in the roots under the salt treatments than WT, indicating that endogenous JA played a role in Na^+^ and K^+^ ion transportation from the roots to shoots, which might be the primary cause of the differential responsibility of roots and shoots of the mutant to salt treatments.

## 4. Materials and Methods

### 4.1. Experimental Material, Planting, and Salt Treatments

JA biosynthesis mutant *opr7opr8,* which carried *opr7-5* and *opr8-2* alleles in *ZmOPR7* and *ZmOPR8,* respectively [41], was crossed with B73 to BC_5_-stage, and the double mutant *opr7-5opr7-5/opr8-2opr8-2* (homozygous for both genes) was selected in the self-segregation population for this study. B73 was used as the WT plant. The original mutant *opr7opr8* was provided by Dr. Michael V. Kolomiets (Texas A&M University, USA).

The experiment was conducted in greenhouses in Nanjing Agriculture University, Nanjing, China. Good-quality seeds of two genotypes were surface-sterilized by 20% bleach solution containing sodium hypochlorite ~5% for 10 min followed by three times of washing with sterilized double-distilled water. The seeds were sown in the sand and grew for 10–12 days in a growth room at 28/25 °C with 16/8 h of day/night cycles and ~180 μmol m^−2^ s^−1^ of illumination.

The seedlings of both genotypes at the V3-stage were transferred to an aerated hydroponic system with full nutrients of modified Hoagland solution, which contained 945 mg/L Ca(NO_3_)_2_4H_2_O, 506 mg/L KNO_3_, 80 mg/L NH_4_NO_3_, 136 mg/L KH_2_PO_4_, 493 mg/L MgSO_4_, and 2.5 mL FeSO_4_. FeSO_4_ was prepared by FeSO4.7H2O + EDTA-Na. The micro-nutrient solution was 0.83 mg/L KI, 6.2 mg/L H_3_BO_3_, 22.3 mg/L MnSO_4_, 8.6 mg/L ZnSO_4_, 0.25 mg/L NaMoO_4_, 0.025 mg/L CuSO_4_, and 0.025 mg/L CaCL_2._

The hydroponic solution was replaced every 3rd day to ensure nutrient enrichment and the pH value of the solution. For each 7 L-hydroponic box, nine seedlings of B73 and nine seedlings of *opr7opr8* were planted. The salt treatments were applied to the plant roots 2–3 days after transfer to the hydroponics boxes by replacing the hydroponic solution. The treatment solutions contained the full nutrients of Hoagland and NaCl. The concentrations of NaCl treatments were 0, 100, 200, and 300 mM. The experiment had three repeats for every concentration of salt treatment.

### 4.2. Analysis of Root and Shoot Elongation

Seven days after salt treatment, the plant’s root and shoot were collected and measured instantly after harvesting. Morphological parameters like primary root elongation, shoot length, fresh shoot weight, fresh root weight, dry root-shoot weight, and plant water content were measured by a ruler and an analytical balance. For dry weight (DW) and water content, the samples were dried in an oven at −80 °C for 48 h and measured by an analytical balance.

### 4.3. Ion Content Profiling

Dry shoot and root tissues of each biological replicate were transferred into digestion tubes (Gerhardt, Brackely, UK), supplemented with 5 mL of concentrated nitric acid (HNO_3_), and then vortexed for 6 h. After cooling, the final volume of each sample was adjusted to 10 mL with distilled water and vortexed. Contents of different ions were measured by an inductively coupled plasma optical emission spectrometer (ICP-OES, Perkin Elmer Optima 2100DV) (College of Life Sciences, Nanjing Agriculture University). Blank samples were prepared by adding 5 mL of concentrated nitric acid to an empty digestion vessel and processed.

### 4.4. Determination of Enzymatic Antioxidants

Fresh leaves and roots (0.5 g) of plants were collected 48 h after salt stress and stored at −80 °C for the determination of various antioxidant enzymes. Tissues were ground in a tissue homogenizer. CAT, POX, APX, SOD, H_2_O_2_, and MDA activities were determined by a chemical assay kit (Nanjing Jiancheng Bioengineering Institute, China). One unit of enzyme activity was defined as an absorbance change of 0.01 units per minute, and each enzyme’s activity was expressed as a unit per milligram of protein. Activities of proline, GR (glutathione reductase), and GST (glutathione S-transferase) were measured according to the kit protocol of the manufacturer. All chemicals were bought from Nanjing Jiancheng Bioengineering Institute. The soluble proline content was calculated in micromoles of proline per gram of fresh weight according to a standard curve.

### 4.5. Photosynthetic Pigments

The photosynthetic pigments leaf chlorophyll a (Chl a) and chlorophyll b (Chl b) were measured. Leaf samples were collected 48 h after salt stress. About 30 mg of leaf segment was incubated with 10 mL of acetone (80%) and kept in the dark for 24 h. The absorbance was measured by spectrophotometry at 645 and 663 nm. Chl a and Chl b contents were calculated using MacKinney equations [81].

### 4.6. Stomatal Imaging and Quantification

Stomatal densities in the maize leaf epidermis were observed after 24 h of salt stress under an optical microscope, Olympus BX53. Stomata on the epidermis layer were imprinted using nail varnish painted on fully expanded leaves at V3-stage plants. The stomatal aperture was calculated using ImageJ software (University of Wisconsin-Madison, Madison, WI, USA). The size was measured in ImageJ from a total of 35 stomata from each genotype of each salt treatment concentration, taken from nine biological replicates. Pore aperture and stomata area were measured from imaged biological replicates. The pore area was calculated from the major axis of the measured aperture length, and the minor axis of the measured aperture width at the center of the pore. The stomatal area was calculated from the axes of the measured guard cell length and the doubled guard cell width at the center of the stoma. The stomata aperture was calculated by dividing the stomata width with the stomata length.

### 4.7. Reactive Oxygen Species Visualization

The reactive oxygen species (ROS) in the root tips were detected using a TCS-SP2 confocal laser scanning microscope (LSCM 780, Zeiss, Leica Lasertechnik GmbH, Heidelberg, Germany). The excitation was set at 488 nm and the emission was at 500–530 nm. The root tissues were collected four hours after the salt treatments and loaded with 20 μM 2′,7′-dichlorofluorescin diacetate (H2DCFDA, Sigma) in a 20 mM HEPES/NaOH buffer (pH 7.5) for 20 min. Samples were then washed with distilled water three times for each 15 min and detected immediately by the confocal microscope. Nine samples were selected per treatment and measured. The experiment was performed at 25 °C. The Root tip sections are imaged under bright field (BF) and green light (480–550 nm) fluorescence (GLF) mode with a confocal microscope. The relative fluorescence production of reactive oxygen species in the root tips was quantified based on 25 overlapping confocal scopes by using ImageJ software.

### 4.8. Gene Expression Analysis

Plant leaves were used for gene expression analysis. The total RNA was isolated with the Trizol method (Sigma-Aldrich, Oakville, Ontario, Canada) under RNase-free conditions. The total RNA was isolated from the leaves of control and salt-stressed plants (200 mM NaCl at 0, 2, 4, 6, 12 24, 48, and 72 h). The integrity of isolated RNA samples was examined spectrophotometrically and by gel electrophoresis. RNA samples were quantified using a NanoDrop 2000C spectrophotometer (Nanodrop Technologies, Wilmington, DE, USA).

To eliminate genomic DNA in the total RNA extracted, total RNA samples were treated by DNase I (DNaseI, Invitrogen, Carlsbad, CA, USA) according to the manufacturer instruction. After DNaseI treatment, total RNA samples were tested for genomic DNA (gDNA) residue by the polymerase chain reaction (PCR) using the primer pair for the maize *actin1* gene. No band of PCR amplification of maize *actin1* indicated that the total RNA sample was free of gDNA residue. First-strand cDNA was synthesized from 1 μg of total RNA following the manufacturer’s instructions. Quantitative PCR (qPCR) was done with a QuantiTect SYBR Green PCR Kit (QIAGEN China Co., Ltd., Shanghai, China) using an Opticon 2 system (Biorad, CFX96 USA). Specific primers for the amplification of target cDNAs were designated by primer-Blast of NCBI (available online: https://www.ncbi.nlm.nih.gov/tools/primer-blast/index.cgi, accessed on 5 December 2019) based on the target gene sequence. The primers are listed in Table S1. The two-way analysis of variance was employed followed by Duncan’s multiple range test to determine the significance of the differences of target gene expression levels among treatments at the level of *p* ≤ 0.05 or *p* ≤ 0.01.

### 4.9. Statistical Analysis

All the treatments were arranged in a completely randomized design. Morphological, physiological, and biochemical data were presented as mean ± SD (standard error). The data were analyzed using a statistical package, Statistic 8.1 (Analytical Software, Tallahassee, FL, USA). The data were subjected to the two-way analysis of variance (ANOVA). Significant differences at levels of significance (* *p* ≤ 0.05; ** *p* ≤ 0.01) are represented by asterisks.

## Supplementary Materials

Supplementary materials can be found at https://www.mdpi.com/1422-0067/20/24/6202/s1. Figure S1, Visualization of stomata in B73 and opr7opr8 mutant. The leaves of B73 and opr7opr8 under control, 100 mM, and 300 mM salt stress were used to image stomata 24 h after application of NaCl under a microscope at 40× magnification; Table S1, Primers used for quantitative real time PCR (qRT-PCR) in the study.

## Abbreviations

FRW	Fresh root weight
FSW	Fresh shoot weight
DRW	Dry root weight
DSW	Dry shoot weight
FSL	Fresh shoot length
FRL	Fresh shoot length
Pro	Proline
MDA	Malondialdehyde
Chl	Chlorophyll
CAT	Catalase
SOD	Superoxide dismutase
APX	Ascorbate peroxidase
