# Supplementary material for: Interruption of Jasmonic Acid Biosynthesis Causes Differential Responses in the Roots and Shoots of Maize Seedlings against Salt Stress
Source: Int J Mol Sci. 2019 Dec 9;20(24):6202. doi: 10.3390/ijms20246202 (PMC6969903; doi:10.3390/ijms20246202)
Supplement: Supplementary file 1 [file ijms-20-06202-s001.pdf]

Appendix

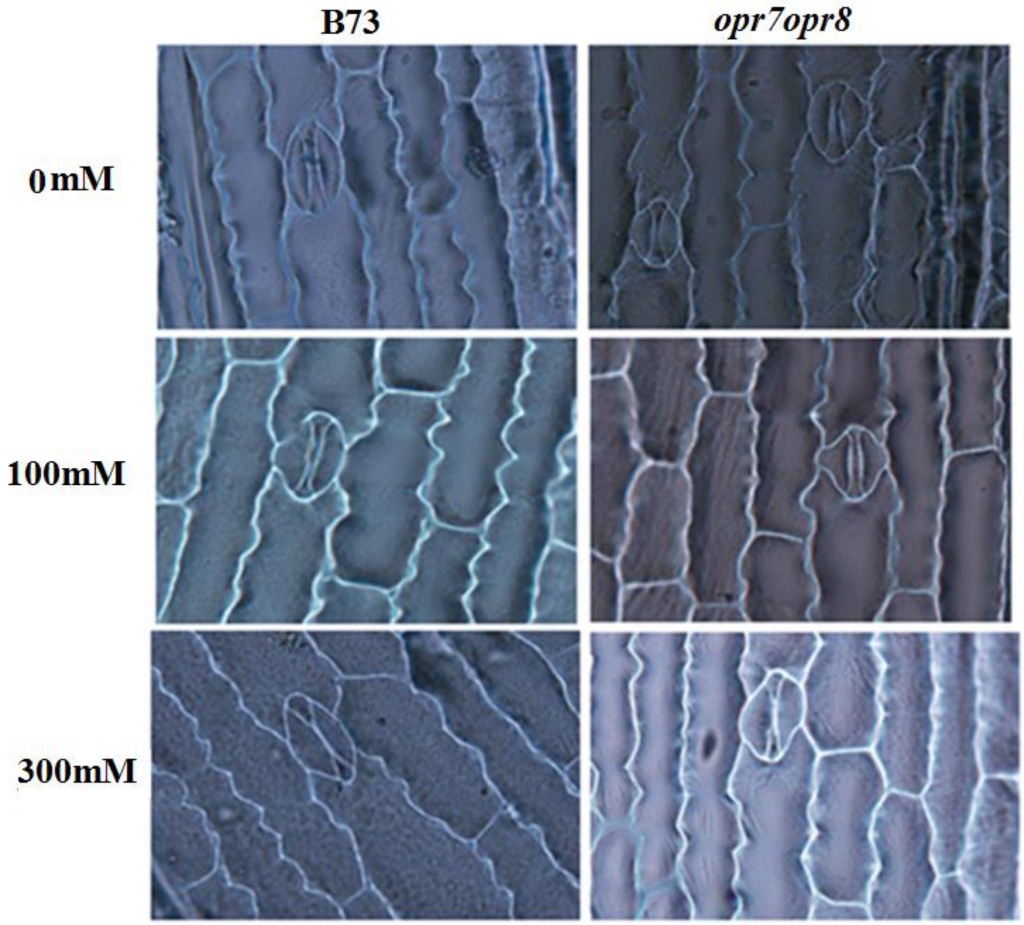

Figure S1 Visualization of stomata in B73 and opr7opr8 mutant. Leaves of B73 and *opr7opr8* under control, 100mM and 300mM salt stress were used to image stomata 24 hours after application of NaCl under microscope at 40x magnification.

Table S1 The primers used for quantitative real time PCR (qRT-PCR) in the study.

Gene name	Forward primer 5'-3'	Reverse primer 5'-3'	Annealing temperature
ZEP1	AATGAAGAGGCTGGTGGCACT	CATAGCATGGACAGAATCACCAAG	58
VP10	ACGAGACAGTAGGAGAGCTCTGTGTC	CAGTGAAAGAAGTTCGTGAGCAAG	59
AO1	AACACAGCGAAAGCATAGATCCA	TAGTGCGTGGGAGATGTGCA	58
Actin	CTGAGGAACTATTCCAGCCTACC	CCACCACTGAGGACAACTTACC	58
NCED 5	CACCACACCGAAAACCCAAC	TGTTGTGGTACACCAGGCCG	59

Primers used to amplify ABA pathway genes in salt stressed leaves of B73 and *opr7opr8* under 200mM NaCl Treatment. *ZEP1*, Zm00001d003513, encoding zeaxanthin epoxidase1; *VP10*, Zm00001d026515, encoding viviparous10; *AO1*, Zm00001d034387, encodeing aldehyde oxidase 1; *NCED 5*, Zm00001d018819, encoding nine-cisepoxycarotenoid dioxygenase 5. *Actin2,* Zm00001d012277.
